# Dataset on quantitative behavioral and EEG of lateral cerebellar nucleus (LCN) electrical stimulation in PTZ-kindled rats

**DOI:** 10.1016/j.dib.2025.111704

**Published:** 2025-05-28

**Authors:** Leonid S. Godlevsky, Mykhailo P. Pervak, Olha S. Yehorenko, Serhii V. Marchenko

**Affiliations:** a Department of Physiology and Biophysics, Odesa National Medical University, Valikhovsky Lane 2, Odesa, 65082, Ukraine; b Department of Simulative Medical Technologies, Odesa National Medical University, Valikhovsky Lane 2, Odesa, 65082, Ukraine

**Keywords:** Lateral cerebellar nucleus, Electrical stimulation, Pentylenetetrazol kindling, Myoclonus, Generalized seizures

## Abstract

The data represented here are in relation to the manuscript (Godlevsky et al., 2025) [1], which quantitatively estimated the effects of the lateral cerebellar nucleus (LCN) electrical stimulation (100 Hz) in rats kindled with pentylenetetrazol (PTZ) daily administration. Both behavioral Racine scale scoring seizure severity and EEG seizure discharges registered in the frontal cortex of rats kindled to the early stage of kindling (not exceeded 2 scored seizure severity) after 9-11 PTZ, and fully developed kindling (4-5 scored seizure severity) after 21 PTZ were quantitatively analyzed. The data in the Tables favors the contribution of LCN to the dynamic of behavioral and EEG seizures, namely, the facilitation of myoclonic seizures at an early stage and the prevention of generalized seizures at the stage of kindled tonic-clonic seizures. Accordingly, all results are presented in primary individual form and quantitatively analyzed, which might better understand LCN’s role in the pathogenesis of chronic epileptic syndrome.

Specifications Table

**Table:** 

Subject	Health Sciences, Medical Sciences & Pharmacology
Specific subject area	Analysis of the Pathogenesis of Pentylenetetrazol-Induced Kindled Seizures as a Model for Chronic Epilepsy
Type of data	Tables, graph
Data collection	In adult male rats (n = 31) were modeled with kindled seizures via PTZ administrations in a dosage of 35.0 mg/kg, i.p. Subdivision of the initial group into subgroups was performed twice: the first one, when two groups were composed by the severity of seizures after the 11^th^ PTZ. Thus, early kindled rats with seizures not exceeding two scored points of severity were used for LCN ES (n = 14). The rest (n = 17) with higher scores for seizure severity composed a group with continued PTZ kindled procedure, and EC LCN started after 21^st^ PTZ administration when fully developed kindling was proved. Blind method of second subdivision of the groups with early and fully developed kindling was used. Each was recomposed into control (sham stimulated, 7 rats for early and 7 for postponed kindling) and experimental (8 and 9 animals for early and postponed kindling correspondently) groups with ES. Data were collected after a five-day course of LCN ES (100 Hz) and testing PTZ (35.0 mg/kg, i.p.) administration. Spikes and ictal discharges were quantified in all subgroups. Only those histologically proven correct localization of stimulative electrodes were used for the analysis.
Data source location	University: Odesa National Medical University City/Town/Region: Odesa, Odesa Region Country: Ukraine
Data accessibility	Repository name: Mendeley Data Data identification number: 10.17632/vn8ccmr8kx Direct URL to data: https://doi.org/10.17632/vn8ccmr8kx.2 Instructions for accessing these data: All data files are available in CSV format in the repository. Each dataset corresponds to a specific experimental table described in this article.
Related research article	Leonid Godlevsky, Mykhailo Pervak, Olha Yehorenko, Serhii Marchenko, Effects of electrical stimulation of the lateral cerebellar nucleus on PTZ-kindled seizures, Epilepsy & Behavior, Volume 167, 2025, 110377. https://doi.org/10.1016/j.yebeh.2025.110377

## 1 Value of the Data

• Data show the role of the lateral cerebellar nucleus in chronic brain epileptisation, namely in myoclonic epilepsy and generalized tonic- clonic seizure pathogenesis.
• Reported data on parameters regimen of stimulation and correspondent outcomes can be used for working out optimal approach to neuromodulation of brain structures in epilepsy.
• Data contributes to resolving contradictions on the cerebellum role in epilepsy, including resistant forms of seizures.
• Data disclosed the potential of the PTZ-induced kindling model as an effective one for neuromodulatory influences investigations.
• The dataset preserves the individual history of each rat throughout the experimental stages, allowing the direct analysis of behavioral and EEG changes without averaging across groups.

## 2 Background

The role of the lateral cerebellar nucleus (LCN) and the cerebellum as a whole in epilepsy manifestations remains controversial [2,3]. LCN destruction causes the facilitation of amygdalar kindling in rats [4], while electrical stimulation (ES) induces both suppression and facilitation of epileptogenesis [1,5].

LCN stimulation has been shown to accelerate motor recovery after stroke — an effect underpinned by the suppression of neuroinflammation in damaged brain tissue [6]. The role of neuroinflammation in PTZ-induced kindled seizures is also established [7,8]. That favored the potential efficacy of LCN ES in this model. Besides, different clinical forms of epilepsy precipitation depending on the stage of kindling development justify exploring this model for LCN ES investigation [5]. Such rationality is advocated by the data on the opposite state of GABA inhibition in the hippocampus — activation at the PTZ-early kindling stage and suppression at the stage of fully developed seizures [9,10].

The data in this study were collected through in vivo animal experimentation aimed at a quantitative assessment of epileptogenesis in PTZ-kindled rats. The dataset reflects the individual nature of behavioral and EEG manifestations in each animal, forming a basis for establishing correlative links and resolving contradictions regarding the role of the LCN in epileptogenesis.

## 3 Data Description

The present dataset comprises the effect of LCN ES (100 Hz) upon behavioral and EEG seizure manifestations at early and fully developed seizures during PTZ-induced kindling development.

The parameters based on the Racine score of seizure severity estimation are listed in Table 1, Table 2 for the early stage of kindling. Table 1 comprises data at the stage of blind subdivision of the group with seizure severity of not more than two scored points into control (not colored lines) and rats with LCN ES (gray color). Correspondingly, Table 2 illustrates behavioral seizures scored in the control group (not colored) and after LCN ES (gray columns). EEG data — the number of spikes per minute averaged for 10-minute intervals — are presented in Table 3, Table 4 for the early kindling stage. The order of rats marked in Table 1 corresponds to the order in Table 2 — control (not colored) and ES LCN (marked with gray color in both tables). Individual data on EEG order presented in Table 3, Table 4 also correspond to the order of data in Table 2.

**Table 1:** Seizure characteristics after the 11th PTZ administration (35.0 mg/kg, i.p.) prior to sham stimulation or LCN ES in the early kindling stage.

Latency of seizures (seconds)		Seizure severity score (Racine scale)
1	76	2
2	63	2
3	91	2
4	113	1
5	65	2
6	76	2
7	90	2
8	105	1
9	62	2
10	60	2
11	73	2
12	117	1
13	84	2
14	95	2
15	72	2

**Table 2:** Seizure severity scored after LCN ES.

	Control (n = 7)		LCN ES (n = 8)	
	Latency of seizures (s)	Score (Racine)	Latency of seizures (s)	Score (Racine)
1	65	2	60	3
2	86	1	62	2
3	115	1	77	2
4	72	2	80	2
5	77	2	65	3
6	60	2	80	2
7	98	1	96	2
8	-	-	84	2

**Table 3:** Number of spikes per minute in the control group. Mean values are presented for each rat.

	10 minutes	20 minutes	30 minutes
1	17	20	18
2	18	25	21
3	14	16	15
4	21	26	18
5	16	17	10
6	13	17	16
7	11	15	19

**Table 4:** Number of spikes per minute in rats with LCN ES.

	10 minutes	20 minutes	30 minutes
1	24	37	40
2	22	48	47
3	20	45	29
4	31	44	32
5	17	29	37
6	13	24	18
7	19	40	27
8	14	30	41

Blind subdivisions in fully developed kindled rats into control (not colored) and LCN ES (gray-colored) subgroups are presented in Table 5. Table 6 presents data on behavioral seizures scored in the control group (not colored) and after LCN ES (gray columns). Table 7 comprises data on the duration of ictal discharges in control and ES LCN groups. The individual order of rats in Table 6, Table 7 corresponds to their order in Table 5.

**Table 5:** Seizure characteristics after the 21st PTZ (35.0 mg/kg, i.p.) before the start of sham stimulation in the control and LCN ES groups.

	Latency of seizures (seconds)	Seizure severity score (Racine scale)
1	85	4
2	72	3
3	58	4
4	67	4
5	94	3
6	64	5
7	79	4
8	53	5
9	68	5
10	62	5
11	87	4
12	64	5
13	96	3
14	70	5
15	65	5
16	56	5

**Table 6:** Seizure severity scored after LCN ES.

	Control - sham stimulated (n=7)		LCN ES (n=9)	
	Latency of seizures (seconds)	Seizure severity score (Racine scale)	Latency of seizures (seconds)	Seizure severity score (Racine scale)
1	73	4	63	4
2	64	4	75	3
3	60	4	60	4
4	90	4	110	2
5	55	5	92	2
6	60	5	53	4
7	79	4	90	3
8	-	-	83	2
9	-	-	60	4

**Table 7:** Duration of ictal potential in control and experimental groups (seconds).

	Control rats (n=7)	Rats with LCN ES (n=9)
1	21	Not registered (technical error)
2	18	-
3	15	9
4	10	-
5	23; 9	-
6	11.5	12
7	12	-
8	-	-
9	-	10

Individual data of rats with LCN ES at the stages of kindling have been illustrated in a data repository (DOI: 10.17632/vn8ccmr8kx).

This paper shows the contribution made by LCN to myoclonus and generalized seizure pathogenesis. Overall, the findings favor the correspondence between behavioral and EEG seizures, which can help avoid errors in identifying kindling stages [11].

**The early stage of kindling — behavioral seizure scoring**

Note: Rows shaded in gray indicate rats assigned to the LCN ES group (n = 8). The rest form the control group (n = 7), as shown in Table 2.

Individual data of each rat listed in Table 1 were recomposed in a blind manner and preserved in the same order in Table 2 for the control group (unshaded) and the LCN ES group (highlighted with grey background).

**Fully developed kindling—EEG seizure quantification**

Individual data for each rat listed in Table 5 were recomposed in a blind manner and preserved in the same order in Table 6, with the control group shown unshaded and the LCN ES group highlighted with a grey background.

## 4 Experimental Design, Materials and Methods

### 4.1 Experimental animals

Experiments were performed on 31 male Wistar rats aged two to three months. Animals were kept under standard conditions: constant temperature (23°C), relative humidity of 60%, a 12-hour dark/light cycle; standard diet and tap water were provided ad libitum. Animals were acclimatized to the laboratory environment for at least seven days before the experiment.

### 4.2 Epilepsy model

Kindled seizures were induced with PTZ (Sigma Aldrich, St. Louis, MO, USA) was given intraperitoneally (i.p.) in a daily dose of 35.0 mg/kg for 21 days. Myoclonus precipitation is associated with SWD. Such myoclonus is pronounced at the feeble level and does not encompass the whole body repeatedly. That is why we addressed severe whole-body myoclonus in the third stage of kindling development.

Hence, the seizure responses were observed over a 30-minute cutoff period and were classified as initially described by Racine: zero: no response; one: vibrissae twitching, mouth, ear, and facial twitching; two: convulsive waves through the body; three: whole body myoclonic jerks, rearing; four: tonic-clonic convulsions with rearings and falling; five: repeated seizures as at stage four, loss of postural control. Seizures were videotaped, and the severity score was determined in a blinded manner.

Seizures were videotaped, and the severity score was determined in a blinded manner.

#### 4.2.1 Criteria for inclusion of rats into observation

The early kindled stage was modeled in rats demonstrating minor manifestations, including myoclonus of muscle groups of extremities and body (one-two scored the severity of seizures). Rats with whole body myoclonus or rearings (three-scored severity) until the 11th PTZ administration composed the group treated with the PTZ for induction fully kindled seizures.

### 4.3 Groups of animals

Seven rats composed the early kindling control group, and seven formed the control group of fully kindled animals (14 rats in total). Detailed study design is schematically represented earlier [1]. Experimental groups subjected to ES trials included eight rats with early kindling and nine with fully kindled seizures (17 rats). Animals were assigned to sham or LCN conditions in a balanced design. Thus, control and experimental groups included animals with an equally proportioned number of rats concerning their seizure severity score (Table 1). Overall, at the stages of operations, kindling development, and performing stimulations, 12 rats were excluded due to the absence of correspondence to inclusion criteria — 5; not stable electrode fixation — 2; death — 2; and the incorrect location of electrodes — 3 rats.

### 4.4 Surgery

In animals, anesthesia was performed with i.p. ketamine administration (100 mg/kg, i.p., Farmak, Ukraine). Rats were placed in a stereotactic apparatus, “SEZh-5” (Kyiv, Ukraine). A 0.5% Novocain solution infiltration (Darnitsa, Ukraine) was used for local anesthesia, and the head was shaved and cleaned with iodine before incision. EEG acquisition and stimulation electrodes were placed about the bregma after skin dissection (2 cm along a middle sagittal axis), and soft tissues were removed from the skull surface. A 1.5-2.0 mm burr hole for EEG acquisition and a 3.0-4.0 mm hole for stimulation electrodes were drilled through the cranium with a standard portable dental drill (Colt 1, Charkov, Ukraine).

The nichrome monopolar electrodes were implanted in the frontal cortex (AP = 1.7; ML = 2.0; DV = 1.0) of both hemispheres, according to the rat brain atlas [12]. The custom-made nichrome electrodes had a diameter of 0.15 mm and an interelectrode distance of 0.25 to 0.30 mm, except for the tip, which was insulated with 25 pm polyesterimide. They were implanted at the left LCN (AP = -10.8, -11.3; ML = 3.5; DV = 6.0). The reference electrode was located in the nasal bones. The electrodes were fixed on the cranial surface with dental cement. 5.0 ml of 0.9% NaCl solution was heated to 35°C and injected i.p. at the end of surgery to prevent dehydration. Penicillin potassium salt (100,000 IU/kg, intramuscular injection) was administered every 12 h during 48 hours post-surgery to prevent infection. Animals were allowed to recover for 10 to 14 days after surgery before observations.

### 4.5 Electrical stimulation

After setting the ES intensity, which does not induce behavioral reactions, stimulations were delivered once per day during the next five days [1]. Stimulation time was 11-12 a.m., and were performed using the electrical stimulator ESU-2 (universal electrical stimulator, FSU), with a 100 Hz impulse frequency, 0.25 ms biphasic pulse duration, 50-100 mcA intensity, and 4.5-5.0 s of ES duration. Sham stimulations were conducted by connecting the animal’s electrodes to the stimulator without delivering an electrical current.

### 4.6 EEG data acquisition and analysis

The analog data were acquired using a computer electroencephalograph DX-5000 (Ukraine), and the data were digitized at a 256 Hz sampling rate. The time constant was 0.1, and the low-pass filter was set at 70 Hz. Shallow frequency and high amplitude (excessive) waves on EEG synchronously appearing in all leads coinciding with behavioral movements were treated as artifacts and excluded from the analysis. The polygraph recordings were analyzed offline visually, and epochs containing artifacts were eliminated.

### 4.7 Visual control of electrode location

All experimental animals were euthanized with Pentobarbital (100.0 mg/kg, i.p.). Upon completion of the experiment, the visual quality control of the electrode placement was ex tempore on the gently removed tissue (no transcardial perfusion). For this purpose, electrocoagulation was performed in the electrode placement area, applying the direct current with an amplitude of 5.0 mA over 30 s and using the electrodes as an anode [1].

### 4.8 Statistics

The statistical package SPSS 20.0 (USA) was used to evaluate the results. The normality of data was checked by the Shapiro-Wilk test. A one-way ANOVA followed by Tukey HSD test was performed to detect seizure latency and the length of ictal discharge difference between groups. Kruskal-Wallis followed with a posthoc Dunn‘s was used for seizure severity and a number of spikes in the poststimulative period. A comparison of seizure severity was performed between three groups: before ES, LCN-stimulated, and control groups. Fisher exact test was used to compare the number of rats with generalized seizures. P-values < 0.05 were considered significant.

## Limitations

Only adult male Wistar rats (n = 31) were used, which may limit the generalizability of findings across sexes and strains.

Ictal discharge durations could not be recorded in some subjects (technical errors), leading to incomplete entries in Table 7.

The PTZ-kindling paradigm reflects one form of chemically induced epileptogenesis and may not capture all features of chronic epilepsy or other seizure models.

The location of stimulative electrodes in different parts of LCN, such as the biggest cerebellar nucleus, varies.

Only a single stimulation frequency (100 Hz) and current range (50–100 μA) were tested; the dataset does not address intensity–response or frequency–response relationships.

## Ethics Statement

All experimental procedures followed the National Institutes of Health guidelines for the care and use of laboratory animals and complied with the European Council Directive of 24 November 1986 for the Care and Use of Laboratory Animals (86/609/EEC). Planning considerations were based on the ARRIVE guidelines and the Basel Declaration (http://www.basel-declaration.org), which emphasizes the principles of the 3Rs (Replacement, Reduction, Refinement). The experimental protocol was approved by the Bioethics Committee of Odesa National Medical University (approval No. 3, dated 14/03/2018).

## CRediT Author Statement

**Leonid S. Godlevsky:** Writing – original draft, Validation, Supervision, Project administration, Conceptualization. **Mykhailo P. Pervak:** Writing – review & editing, Visualization, Software, Data curation. **Olha S. Yehorenko:** Writing – review & editing, Visualization, Formal analysis, Data curation. **Serhii V. Marchenko:** Writing – review & editing, Visualization, Data curation.

## Data Availability

Mendeley DataLATERAL CEREBELLAR NUCLEUS (LCN) CONTRIBUTION TO MYOCLONUS PATHOGENESIS IN PTZ-KINDLED RATS (Original data)
